# Effectiveness and safety of GnRH antagonist originator and generic in real-world clinical practice: a retrospective cohort study

**DOI:** 10.3389/fendo.2024.1358278

**Published:** 2024-06-14

**Authors:** Mingzhu Cao, Yuqi Hu, Jiaqi Xiao, Sichen Li, Yanshan Lin, Jianqiao Liu, Haiying Liu

**Affiliations:** ^1^ Department of Obstetrics and Gynecology, Center for Reproductive Medicine, Guangdong Provincial Key Laboratory of Major Obstetric Diseases, Guangdong Provincial Clinical Research Center for Obstetrics and Gynecology, Guangdong-Hong Kong-Macao Greater Bay Area Higher Education Joint Laboratory of Maternal-Fetal Medicine, The Third Affiliated Hospital of Guangzhou Medical University, Guangzhou, China; ^2^ Department of Clinical Laboratory Medicine, The Third Affiliated Hospital of Guangzhou Medical University, Guangzhou, China; ^3^ Department of Clinical Medicine, The Third Clinical School of Guangzhou Medical University, Guangzhou, China; ^4^ Department of Clinical Medicine, The Nanshan College of Guangzhou Medical University, Guangzhou, China

**Keywords:** GnRH antagonist, generic, live birth rate, Cetrotide^®^, Ferpront^®^

## Abstract

**Objective:**

This study aims to determine whether the live birth rates were similar between GnRH antagonist original reference product Cetrotide^®^ and generic Ferpront^®^, in gonadotropin-releasing hormone (GnRH) antagonist protocol for controlled ovarian stimulation (COS).

**Methods:**

This retrospective cohort study investigates COS cycles utilizing GnRH antagonist protocols. The research was conducted at a specialized reproductive medicine center within a tertiary care hospital, spanning the period from October 2019 to October 2021. Within this timeframe, a total of 924 cycles were administered utilizing the GnRH antagonist originator, Cetrotide^®^ (Group A), whereas 1984 cycles were undertaken using the generic, Ferpront^®^ (Group B).

**Results:**

Ovarian reserve markers, including anti-Mullerian hormone, antral follicle number, and basal follicular stimulating hormone, were lower in Group A compared to Group B. Propensity score matching (PSM) was performed to balance these markers between the groups. After PSM, baseline clinical features were similar, except for a slightly longer infertile duration in Group A versus Group B (4.43 ± 2.92 years vs. 4.14 ± 2.84 years, *P =* 0.029). The duration of GnRH antagonist usage was slightly longer in Group B than in Group A (6.02 ± 1.41 vs. 5.71 ± 1.48 days, *P <* 0.001). Group B had a slightly lower number of retrieved oocytes compared to Group A (14.17 ± 7.30 vs. 14.96 ± 7.75, *P =* 0.024). However, comparable numbers of usable embryos on day 3 and good-quality embryos were found between the groups. Reproductive outcomes, including biochemical pregnancy loss, clinical pregnancy, miscarriage, and live birth rate, did not differ significantly between the groups. Multivariate logistic regression analyses suggested that the type of GnRH antagonist did not independently impact the number of oocytes retrieved, usable embryos, good-quality embryos, moderate to severe OHSS rate, clinical pregnancy, miscarriage, or live birth rate.

**Conclusion:**

The retrospective analysis revealed no clinically significant differences in reproductive outcomes between Cetrotide^®^ and Ferpront^®^ when used in women undergoing their first and second COS cycles utilizing the GnRH antagonist protocol.

## 1 Introduction

Infertility, defined as the failure to conceive within a year of unprotected sexual activity, remains a persistent global challenge (1). Assisted reproductive technologies (ART), such as *in vitro* fertilization (IVF) and intra-cytoplasmic sperm injection (ICSI), offer effective solutions for infertility (1). Controlled ovarian stimulation (COS) using exogenous gonadotropins stands as a critical step in ART, enabling the recruitment of a sufficient number of fertilizable oocytes and subsequent embryo formation. The two most widely used protocols in COS are the Gonadotropin Releasing Hormone (GnRH) antagonist and GnRH agonist protocols.

The GnRH antagonist protocol presents several advantages over the GnRH agonist protocol, including a shorter duration of antagonist treatment, reduced gonadotropin (Gn) stimulation, lowered risk of ovarian hyperstimulation syndrome (OHSS), and absence of flare-up effects and low estrogen impact (2, 3). GnRH antagonists inhibit luteinizing hormone (LH) release directly and swiftly by competitively binding to GnRH receptors in the pituitary (3). Notable antagonists used in clinics include cetrorelix and ganirelix.

Cetrotide^®^, also known as Cetrorelix acetate injection (patent expired in April 2019), is a synthetic decapeptide recognized for its stability, minimal variability, high bioavailability, and efficacy in preventing premature LH surges during COS in females (4, 5). It was the first GnRH antagonist introduced in clinical settings (6). Ferpront^®^ (Ferring Pharmaceuticals, China) is a cetrorelix generic developed to emulate Cetrotide^®^’s physicochemical properties (7). In December 2018, Ferpront^®^ received authorization from the Chinese Center for Drug Evaluation (8). Pre-clinical studies have demonstrated the safety and pharmacokinetics of Ferpront^®^ compared to Cetrotide^®^7.

Despite their shared similar active pharmaceutical ingredients, there remains a lack of comparative studies evaluating the efficacy and safety between Ferpront^®^ and Cetrotide^®^. To address this gap, the current retrospective cohort study aims to investigate the clinical efficacy and safety of Ferpront^®^ as a generic of Cetrotide^®^ in infertile women undergoing COS with the GnRH antagonist protocol.

## 2 Materials and methods

### 2.1 Study population

The study was a retrospective, single-center investigation conducted at the Guangzhou Medical University Third Affiliated Hospital. Notably, this hospital stands as one of the largest reproductive medicine centers in Southern China, performing nearly 10,000 ART cycles annually. The study protocol received approval from the ethical committee (approval number: 2023–121). Comprehensive clinical records, encompassing detailed demographic and treatment-related data, were extracted from the hospital’s database for analysis. Clinical records from infertile couples undergoing IVF or ICSI between October 2019 and October 2021 were screened for eligibility criteria. Included participants met specific criteria: utilization of the GnRH antagonist protocol for COS, involvement in either the first or second COS cycle, females aged 20–40, and use of either Cetrotide^®^ (Group A) or Ferpront^®^ (Group B) as the GnRH antagonist. Exclusion criteria comprised compromised endometrial conditions, severe endometriosis, repeated miscarriages or implantation failures, pre-implantation genetic testing, fertility preservation, Micro-TESE sperm retrieval, oocyte or embryo banking cycles, and severe systemic diseases potentially impacting reproductive outcomes. Consecutive participants fulfilled the inclusion and exclusion criteria were included for further analysis.

### 2.2 Ovarian stimulation protocols and embryo transfer

Cycles with GnRH antagonist protocols were included, incorporating several types of gonadotropins (Gn), including recombinant follicular stimulating hormone (Gonal-F^®^, Merck & Co., Germany; Puregon^®^, Organon & Co., USA), urine FSH (LiShengBao^®^, Livzon Pharm, China), and human menopausal gonadotropin (LeBaoDe^®^, Livzon Pharm, China) for COS. In the fixed protocol, the GnRH antagonist—either 0.25 mg of Cetrotide^®^ or Ferpront^®^—was administered daily on day 5 of Gn administration. In the flexible protocol, the initiation of 0.25 mg GnRH antagonist occurred upon meeting at least one of the following criteria: 1) the dominant follicle reached an average diameter of 12 mm, 2) serum E2 levels were > 550–1400 pmol/L (150–400 pg/ml), 3) serum LH was elevated more than 2 times the baseline level or LH ≥ 10 IU/L.

GnRH antagonist administration continued until the day of ovulation trigger. Regular monitoring of follicle development through transvaginal ultrasound and serum FSH, LH, estradiol (E2), and progesterone (P) levels was performed. The ovulation trigger was administered if there were at least 2 leading follicles with a mean diameter of 18 mm or at least 3 leading follicles ≥ 17 mm, using of recombinant human chorion gonadotropin (Ovidrel^®^, Merck & Co., Germany), 2000 to 10000 IU of urine HCG (Livzon Pharm, China), or 0.2 mg of GnRHa (Decapeptyl^®^, Ferring Pharmaceuticals, Switzerland). Transvaginal oocytes recollection was arranged approximately 34 to 36 hours after the trigger, and fertilization with IVF or ICSI was performed based on semen quality.

The freeze-all policy was applied under several conditions: 1) if more than 20 oocytes were retrieved, 2) serum E2 levels were ≥ 18350 pmol/L on the trigger day, 3) other medical conditions deemed unsuitable for fresh embryo transfer as determined by physicians, 4) personal reasons prohibiting fresh embryo transfer. One or two cleavage stage embryos or blastocyst embryos were transferred either 3 or 5 days following oocytes pick-up (OPU) day, and the remaining usable embryos were vitrified. Embryo grading was conducted based on fragmentation levels (9) (Grade I: < 5%, Grade II: 5–20%, Grade III: 20–50%, Grade IV: < 50%). An embryo with good quality on day 3 was defined as 7–9 cells with < 20% cellular debris and uniformity in cell size. Blastocyst quality was evaluated based on the Gardner scoring system for trophectoderm and inner cell mass scores. Routine luteal phase support with dydrogesterone 20 mg/day (Duphaston^®^, Abbott Laboratories, USA), 90 mg/day vaginal progesterone gel (Crinone^®^, Merck, Germany), or 0.2 g/day of vaginal progesterone capsule (Utrogestan^®^, Besins Healthcare, Monaco) was administered post-oocyte retrieval and continued after fresh embryo transfer. Pregnancy was evaluated through serum HCG testing 14 days following embryo transfer and transvaginal ultrasound examination approximately 4 weeks after embryo transfer.

### 2.3 Outcomes measured

The study’s primary endpoint was the live birth rate per embryo transfer cycle, defined as the delivery of live newborns after 28 weeks of gestation. The birth of twins or triplets was considered as one live birth. Secondary endpoints included: 1) the number of retrieved oocytes, 2) the number of usable embryos on day 3, 3) the number of good quality embryos, 4) clinical pregnancy rate, and 5) spontaneous miscarriage rate. Biochemical pregnancy was identified by detecting serum HCG > 10 mIU/ml two weeks post-embryo transfer, while clinical pregnancy was confirmed by observing an intrauterine gestational sac via ultrasonography around 6 weeks of gestation. Spontaneous miscarriages were characterized by pregnancy losses with detectable intrauterine gestational sacs before 28 weeks of gestation. Biochemical pregnancy rate, clinical pregnancy rate, and live birth rate was calculated as the percentage of cycles meeting these criteria out of cycles with fresh embryo transfer. The spontaneous miscarriage rate was determined as the proportion of cycles experiencing spontaneous miscarriage among those resulting in clinical pregnancy.

Safety outcomes were measured by evaluating the incidence of moderate/severe OHSS. The diagnosis criteria followed recommendations from a consensus of Chinese experts (10). Moderate OHSS was identified by the presence of abdominal discomfort, nausea, vomiting, diarrhea; ovarian enlargement (8–12 cm) and ascites detected through ultrasound; and specific laboratory findings including a hematocrit < 0.45 and elevated leukocyte count (10–15 × 10^9^/L). Severe OHSS presented symptoms such as severe nausea, vomiting, dyspnea, significant abdominal pain, oliguria or anuria (< 300 ml/d or < 30 ml/h), rapid weight gain (> 1 kg/24 h), enlarged ovaries (> 12 cm) with sonographic evidence of tension ascites, pleural effusion, vascular embolism, low blood pressure, or low central venous pressure. Additionally, it included elevated hematocrit (> 0.45), increased leukocyte levels (> 15 × 10^9^/L), hyperkalemia (potassium >5 mmol/L), hyponatremia (sodium < 135 mmol/L), impaired renal function (creatinine > 1.0 g/L), and altered liver function (increased levels of glutamic oxaloacetic transaminase and glutamic pyruvic transaminase).

### 2.4 Statistical analysis

In the current study, Cetrotide^®^ was used as the reference medication. All statistical analyses were carried out with SPSS (version 22.0, IBM Inc., US). Quantitative variables with a normal distribution were described as mean ± standard deviation (SD) and compared using Student’s t-test, while those with a skewed distribution were depicted as median (25th and 75th quartiles) and compared with the Mann-Whitney U test. Comparisons of frequencies and proportions were performed using the Chi-squared test.

Several baseline clinical parameters, such as baseline follicle-stimulating hormone (FSH), anti-müllerian hormone (AMH), and antral follicle count (AFC) differed significantly between Group A and Group B. To minimize the influence of these confounders, propensity score matching (PSM) was conducted to align these parameters. The two groups were matched 1:1 using nearest neighbor matching. The standardized mean difference (SMD) before and after PSM was calculated and presented in **Supplementary Table 1**, showing a reduced SMD after matching to less than 0.1, considered balanced (11).

To determine if the type of GnRH antagonist independently impacted various reproductive outcomes, multivariate logistic regression analyses were conducted with these outcomes as dependent factors before and after PSM. Possible confounders, including female age, duration of infertility, infertility factors, AMH, AFC, baseline FSH, BMI, duration and dosage of Gn and GnRH antagonist, number of oocytes collected, and trigger type, were included in the multivariable logistic regression before PSM. After PSM, additional potential confounders included in the analyses were female age, duration of infertility, serum AMH, AFC, BMI, duration and total dosage of Gn and GnRH antagonist, and the number of oocytes retrieved. The likelihoods of reproductive outcomes were displayed as adjusted odds ratios (OR) with their 95% confidence intervals (95% CI). Multiple linear regressions using a stepwise selection approach were utilized in a multivariate statistical model to assess the impact of GnRH antagonist type on the number of oocytes retrieved, usable embryos, and good-quality embryos. A significance level of less than 0.05 was considered statistically significant for all analyses.

## 3 Results

### 3.1 Baseline clinical characteristics

**Figure 1:**
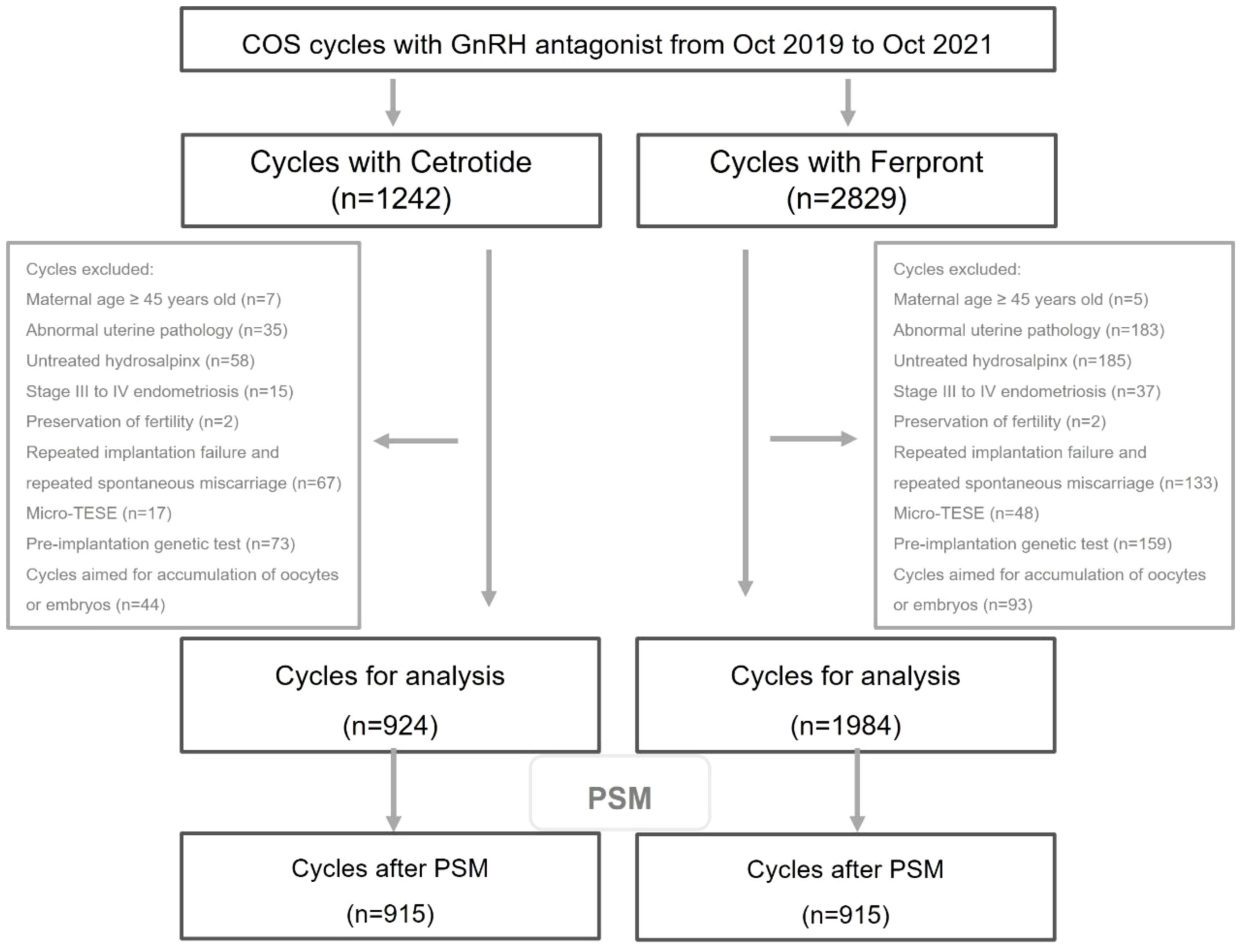
Flow chart of cycles inclusion and exclusion.

Overall, 2908 cycles with the first or second cycles of GnRH antagonist protocol for COS were included and further divided into two groups based on the type of GnRH antagonist used (n=924 for Group A with Cetrotide^®^ and n=1984 for Group B with Ferpront^®^). The flow chart depicting data collection was presented in **Figure 1**.

As presented in **Table 1**, detailed participants’ baseline characteristics, infertile factors in Group B slightly differed from those in Group A. Notably, several ovarian reserve tests, including serum AMH levels, AFC, and baseline FSH levels, were significantly lower in Group B than in Group A. Considering the substantial clinical value of ovarian reserve markers, balancing these markers using PSM was performed. As revealed in **Supplementary Table 1**, the SMD in the ovarian reserve markers after PSM was noticeably less than before PSM, thus achieving a well-balanced status for the ovarian reserve markers post-PSM. After PSM, all other parameters were comparable between the two groups, except for the infertile duration, which was slightly higher in Group A than in Group B, but with minimal clinically substantive significance.

**Table 1:** Comparison of basic characteristics.

	Before PSM				After PSM			
	Group A	Group B	t/χ^2^	*P* value	Group A	Group B	t/χ^2^	*P* value
n	924	1984			915	915		
Female age	31.45 ± 4.37	31.51 ± 4.43	-0.302	0.762	31.47 ± 4.36	31.25 ± 4.46	1.066	0.287
Male age	33.49 ± 5.26	33.72 ± 5.30	-1.088	0.277	33.49 ± 5.23	33.43 ± 5.34	0.234	0.815
Infertile duration (years)	4.44 ± 2.93	4.24 ± 2.89	1.784	0.075	4.43 ± 2.92	4.14 ± 2.84	2.19	0.029
Infertile type			0.831	0.362			0.179	0.673
Primary infertility	509/55.09%	1057/53.28%			504/55.08%	496/54.10%		
Secondary infertility	415/44.91%	927/46.72%			411/44.92%	420/45.90%		
Infertile factors			21.568	0.001			10.97	0.052
Male factor	190/20.56%	404/20.36%			188/20.55%	171/18.69%		
Tubal/pelvic factor	301/32.58%	735/37.05%			298/32.57%	332/36.28%		
Endometriosis	14/1.52%	5/0.25%			14/1.53%	3/0.33%		
Ovulation disorder	93/10.06%	184/9.27%			92/10.05%	91/9.95%		
Mixed factors	236/25.54%	452/22.78%			234/25.57%	218/23.83%		
Unexplained	90/9.74%	204/10.28%			89/9.73%	100/10.93%		
BMI (kg/m^2^)	22.37 ± 3.42	22.28 ± 3.26	0.68	0.496	22.36 ± 3.40	22.20 ± 3.22	1.08	0.28
AMH (ng/ml)	5.59 ± 4.13	5.22 ± 3.72	2.459	0.014	5.59 ± 4.13	5.48 ± 3.89	0.559	0.576
Basal FSH level (IU/L)	6.11 ± 2.39	5.91 ± 2.11	2.247	0.025	6.07 ± 2.29	6.03 ± 2.28	0.423	0.672
AFC (n)	22.97 ± 10.17	22.20 ± 9.48	1.944	0.047	22.97 ± 10.17	22.99 ± 10.28	-0.053	0.958
Source of sperm			0.624	0.43			1.028	0.311
Husband’s	895/96.86%	1932/97.38%			887/96.94%	894/97.70%		
Sperm bank	29/3.14%	52/2.62%			28/3.06%	21/2.30%		

PSM, propensity score matching; BMI, body mass index; AMH, anti-Mullerian hormone; FSH, follicular stimulating hormone; AFC, antral follicle counting.

### 3.2 Ovarian stimulation outcomes and embryological results

Ovarian stimulation outcomes and embryological results were presented in **Table 2**. The total dose and duration of Gn, the proportion of urinary Gn, and the duration of GnRH antagonist were significantly higher in Group B compared to those in Group A. There were fewer cycles with E2 > 18350 pmol/L on trigger day in Group B (13.05% vs. 21.65%, *P <* 0.001), and significantly more cycles with HCG for trigger (71.77% vs. 64.39%, *P <* 0.001), and more fresh embryo transfers (54.00% vs. 61.44%, *P <* 0.001) observed in Group B, possibly due to differences in ovarian reserve tests. After PSM for balancing ovarian reserve tests, the previously mentioned differences were minimal, although the duration of GnRH antagonist in Group B was significantly longer than that in Group A, and more urinary Gn was administered in Group B compared to Group A (*P <* 0.001). Before PSM, serum levels of LH and P on trigger day were slightly lower in Group B compared to those in Group A, which remained comparable after PSM. In Group A, more cycles had higher E2 levels (E2 > 18350 pmol/L) on trigger day than those in Group B (21.65% vs. 13.05%), and less cycles had low levels of E2 (E2 < 3670 pmol/L) than those in Group B (1.95% vs. 4.74%), and this trend remined even after PSM (*P* < 0.001). Premature LH surge poses a recognized risk in GnRH antagonist protocols and is a crucial parameter under assessment. While various studies present differing specifics, most commonly cite the LH threshold at LH ≥ 10 IU/L (12). Remarkably, in this study, instances where LH ≥ 10 IU/L were so rare that they were negligible and did not require attention or intervention. However, we did notice a reduced number of oocytes retrieved in Group B than in Group A (13.96 ± 7.19 vs. 14.97 ± 7.76 oocytes, *P =* 0.001), and this difference persisted even after PSM (14.17 ± 7.30 vs. 14.96 ± 7.75 oocytes, *P =* 0.024). The number of usable embryos and good-quality embryos was comparable between the two groups before and after PSM.

**Table 2:** Comparison of cycle characteristics.

	Before PSM				After PSM			
	Group A	Group B	t/Z	*P* value	Group A	Group B	t/Z	*P* value
Cycle number	924	1984			915	915		
COS cycle number			0.096	0.757			0.179	0.672
First cycle	751/81.28%	1622/81.75%			744/81.31%	751/82.08%		
Second cycle	173/18.72%	362/18.25%			171/18.69%	164/17.92%		
Gn type			48.317	< 0.001			41.322	< 0.001
Recombinant	781/84.52%	1637/82.51%			775/84.70%	756/82.62%		
Urinary	44/4.76%	227/11.44%			42/4.59%	106/11.58%		
Combined	99/10.71%	120/6.05%			98/10.71%	53/5.79%		
Gn starting dose	169.16 ± 56.53	169.34 ± 57.53	-0.079	0.937	168.99 ± 56.35	168.70 ± 56.88	0.108	0.914
Gn total dose	1500 (1200, 2025)	1500 (1200, 2025)	-2.775	0.006	1500 (1200, 2100)	1500 (1200, 2025)		0.111
Gn duration	9 (8,10)	9 (8, 10)	-2.724	0.006	9 (9, 10)	9 (8, 10)		0.107
GnRHant duration	5.72 ± 1.47	6.01 ± 1.42	-5.162	< 0.001	5.71 ± 1.48	6.02 ± 1.41	-4.521	< 0.001
GnRHant dose	1.52 ± 0.47	1.54 ± 0.43	-1.137	0.256	1.52 ± 0.47	1.55 ± 0.43	-1.164	0.245
LH level on trigger day (IU/L)	1.54 (0.96, 2.66)	1.32 (0.83, 2.21)	-4.585	< 0.001	2.10 ± 1.85	1.93 ± 3.39	1.258	0.208
P level on trigger day (nmol/L)	2.40 (1.75, 3.50)	2.30 (1.60, 2.10)	-4.115	< 0.001	2.78 ± 1.50	2.63 ± 1.69	1.841	0.066
E2 level on trigger day (pmol/L)			45.287	< 0.001			24.688	< 0.001
< 3670	18/1.95%	94/4.74%			18/1.97%	46/5.03%		
3670–18350	581/62.88%	1361/68.60%			577/63.06%	615/67.21%		
> 18350	200/21.65%	259/13.05%			196/21.42%	135/14.75%		
Type of trigger			18.67	< 0.001			0.33	0.848
HCG	595/64.39%	1424/71.77%			591/64.59%	586/64.04%		
GnRHa	219/23.70%	400/20.16%			217/23.72%	214/23.39%		
Dual trigger	110/11.90%	160/8.06%			107/11.69%	115/12.57%		
Freeze-all cycles			0.161	0.689			0.639	0.424
Reasons for freeze-all								
OHSS risk	349/82.12%	621/81.18%			344/81.90%	310/79.69%		
Others	76/17.88%	144/18.82%			76/18.10%	79/20.31%		
OPU number in COS cycle	14.97 ± 7.76	13.96 ± 7.19	4.456	0.001	14.96 ± 7.75	14.17 ± 7.30	2.255	0.024
Fertilization type			2.024	0.364			0.53	0.767
IVF	724/78.35%	1514/76.31%			717/78.36%	705/77.05%		
ICSI	154/16.67%	374/18/85%			162/17.70%	174/19.01%		
IVF+ICSI	46/4.87%	96/4.84%			36/3.93%	36/3.93%		
Fertilization rate (%)	76.21 ± 20.95	77.68 ± 19.95	-1.812	0.07	76.31 ± 20.92%	77.51 ± 20.17	-1.251	0.211
Cleavage rate (%)	74.96 ± 21.02	76.41 ± 20.13	-1.796	0.073	75.04 ± 21.00%	76.26 ± 20.31	-1.258	0.209
Number of 2PN embryo(s)	7.93 ± 5.04	7.66 ± 4.66	1.409	0.159	7.92 ± 5.03	7.75 ± 4.72	0.767	0.443
Number of usable embryos (D3)	6.22 ± 4.55	5.98 ± 4.09	1.422	0.155	6.23 ± 4.54	6.18 ± 4.15	0.226	0.821
Number of good quality embryo	2.20 ± 2.28	2.18 ± 2.16	0.206	0.836	1.34 ± 0.48	1.38 ± 0.49	-0.483	0.629
Cycles with fresh embryo transfer	499/54.00%	1219/61.44%	14.423	< 0.001	495/54.10%	526/57.49%	2.129	0.145
Endometrial thickness	10.39 ± 1.98	10.46 ± 1.96	-0.786	0.432	10.40 ± 1.98	10.58 ± 1.99	-1.744	0.081
Number of embryos for ET			1.963	0.161			1.815	0.178
N = 1	327/65.53%	755/61.94%			325/65.66%	324/61.60%		
N = 2	172/34.47%	464/38.06%			180/34.34%	202/38.40%		
Embryo stage			0.003	0.956			0.025	0.874
Cleavage	329/65.93%	802/65.79%			327/66.06%	345/65.59%		
Blastocyst	170/34.07%	417/34.21%			168/33.94%	181/34.41%		

PSM, propensity score matching; COS, controlled ovarian stimulation; Gn, gonadotropin; LH, luteinizing hormone; P, progesterone; E2, estrogen; HCG, human chorionic gonadotropin; GnRHa, gonadotropin releasing hormone agonist; OHSS, ovarian hyper-stimulation syndrome; OPU, oocytes pick up; IVF, in vitro fertilization; ICSI, intra-cytoplasmic sperm injection; 2PN, 2 pronucleus; D3, day 3; ET, embryo transfer.

### 3.3 Reproductive and safety outcomes

Reproductive outcomes, such as implantation rate, biochemical pregnancy loss, clinical pregnancy, spontaneous miscarriage, multiple pregnancy, and live birth rate, along with the safety outcome, moderate to severe OHSS rate, were presented in **Table 3**. Notably, no adverse events were reported. The table revealed similar reproductive and safety outcomes between the two groups before and after PSM.

**Table 3:** Comparison of reproductive outcomes.

	Before PSM				After PSM			
	Group A	Group B	χ^2^/t	P	Group A	Group B	χ^2^/t	P
Implantation	42.47% (285/671)	41.53% (699/1683)	0.175	0.676	42.56% (283/665)	40.38% (294/728)	0.676	0.411
Biochemical pregnancy	4.01% (20/499)	4.76% (58/1219)	0.460	0.498	4.04% (20/495)	5.13% (27/526)	0.693	0.405
Clinical pregnancy	51.70% (258/499)	50.29% (613/1219)	0.284	0.594	51.72% (256/495)	49.43% (260/526)	0.534	0.465
Miscarriage	13.19% (34/258)	12.89% (79/613)	0.014	0.907	12.89% (33/256)	11.54 (30/260)	0.220	0.639
Live birth	43.09% (215/499)	42.33% (516/1219)	0.083	0.773	43.23% (214/495)	41.63% (219/526)	0.266	0.903
Multiple pregnancy	12.02% (31/258)	15.01% (92/613)	1.341	0.247	12.11% (31/256)	13.85% (36/260)	0.344	0.557
Birth weight of newborns (kg)	3.00 ± 0.53	3.00 ± 0.53	0.078	0.938	3.00 ± 0.53	3.00 ± 0.52	-0.546	0.585
Birth height of newborns (cm)	49.07 ± 2.66	48.95 ± 2.63	0.529	0.597	49.09 ± 2.65	49.15 ± 2.74	-0.258	0.797
Malformation of newborns	0.93% (2/215)	0.78% (4/516)	/	1.000	0.93% (2/214)	0.91% (2/219)	/	1.000
Moderate/severe OHSS rate	3.57% (33/924)	2.87% (57/1984)	1.025	0.311	3.61% (33/915)	2.51% (23/915)	1.622	0.203

PSM, propensity score matching; OHSS, ovarian hyper-stimulation syndrome.

### 3.4 Multivariate regression analyses

After adjusting for several confounders, the multivariate regression analyses in **Table 4** found that the types of GnRH antagonists were not independent factors influencing the number of oocytes retrieved, usable embryos, and good-quality embryos on day 3, as well as multiple reproductive and safety outcomes before and after PSM.

**Table 4:** Multivariate regression analysis of the impact of GnRH antagonist type.

	Before PSM			After PSM		
	Coefficient	t	*P*	Coefficient	t	P
OPU number	-0.002	-0.126	0.900	-0.002	-0.105	0.917
Number of usable embryos	0.008	0.429	0.668	0.033	1.412	0.158
Number of good quality embryos	0.018	0.942	0.346	0.038	1.566	0.117
	Wald value	95% CI	*P*	Wald value	95% CI	*P*
OHSS	1.181	0.211, 1.561	0.277	3.309	0.958, 3.174	0.069
Clinical pregnancy	0.363	0.729, 1.182	0.547	1.252	0.647, 1.126	0.263
Live birth	0.001	0.787, 1.279	0.980	0.321	0.697, 1.220	0.571
Multiple pregnancy	3.642	0.987, 2.732	0.056	2.134	0.853, 2.967	0.144
Miscarriage	0.182	0.542, 1.481	0.669	0.402	0.443, 1.515	0.526

PSM, propensity score matching; OPU, oocytes pick up; OHSS, ovarian hyper-stimulation syndrome; 95% CI, 95% confidential interval.

### 3.5 Subgroup analysis of fixed and flexible protocol of GnRH antagonist

The subgroup analysis of both fixed and flexible protocol of GnRH antagonist as demonstrated in **Table 5** found no obvious differences of reproductive outcomes between the two groups. Multivariate regression analysis of fixed and flexible protocol as shown in **Table 6** further confirmed that the type of GnRH antagonist had no independent impact on the number of oocytes retrieved, usable embryos, and good-quality embryos, as well as reproductive and safety outcomes regardless of before or after PSM.

**Table 5:** Subgroup analysis of reproductive outcomes from fixed and flexible protocol of GnRH antagonist.

	Before PSM				Before PSM			
Fixed protocol	Group A	Group B	χ^2^	P	Group A	Group B	χ^2^	P
N	342	706			341	328		
Implantation	40.96% (111/271)	39.18% (250/638)	0.250	0.617	40.96% (111/271)	40.81% (111/282)	0.147	0.702
Biochemical pregnancy	2.99% (6/201)	4.54% (21/463)	0.864	0.353	2.99% (6/201)	4.46% (9/202)	0.608	0.436
Clinical pregnancy	50.25% (101/201)	47.95% (222/463)	0.297	0.586	50.25% (101/201)	47.52% (96/202)	0.299	0.584
Miscarriage	13.86% (14/101)	9.91% (22/222)	1.094	0.295	13.86% (14/101)	6.25% (6/96)	3.126	0.077
Live birth	40.30% (81/201)	39.96% (185/463)	0.007	0.934	40.30% (81/201)	42.57% (86/202)	0.215	0.643
Moderate/severe OHSS rate	4.09% (14/342)	2.55% (18/706)	1.855	0.173	3.81% (13/341)	1.83% (6/328)	2.383	0.123
multiple pregnancy	11.88% (12/101)	13.06% (29/222)	0.087	0.767	11.88% (12/101)	16.67% (16/96)	0.924	0.336
Flexible protocol								
N	582	1278			574	587		
Implantation	43.50% (174/400)	42.97% (449/1045)	0.924	0.336	43.65% (172/394)	41.26% (184/446)	0.493	0.483
Biochemical pregnancy	4.70% (14/298)	4.89% (37/756)	0.018	0.894	4.76% (14/294)	5.56% (18/324)	0.198	0.657
Clinical pregnancy	52.68% (157/298)	51.72% (391/756)	0.080	0.778	52.72% (155/294)	50.62% (164/324)	0.273	0.601
Miscarriage	12.74% (20/157)	14.58% (57/391)	0.314	0.575	12.26% (19/155)	14.63% (24/164)	0.386	0.535
Live birth	42.62% (127/298)	41.93% (317/756)	0.924	0.336	44.22% (130/294)	41.05% (133/324)	0.633	0.426
Moderate/severe OHSS rate	3.26% (19/582)	3.05% (39/2178)	0.06	0.806	3.31% (19/574)	2.90% (17/587)	0.166	0.684

PSM, propensity score matching; N, number; OHSS, ovarian hyper-stimulation syndrome.

**Table 6:** Multivariate regression analysis of fixed and flexible protocol of GnRH antagonist.

	Before PSM			After PSM		
Fixed protocol	Coefficient	t	*P*	Coefficient	t	P
OPU number	0.003	0.126	0.900	-0.002	-0.061	0.951
Number of usable embryos	-0.020	-0.780	0.436	0.008	0.237	0.813
Number of good quality embryos	0.029	0.968	0.333	0.039	1.037	0.300
	Wald value	95% CI	*P*	Wald value	95% CI	*P*
OHSS	0.759	0.097, 2.451	0.384	2.633	0.837, 6.637	0.105
Clinical pregnancy	0.486	0.626, 1.250	0.486	0.388	0.581, 1.325	0.877
Live birth	0.001	0.700, 1.417	0.982	3.531	0.958, 7.604	0.060
Multiple pregnancy	0.189	0.560, 2.485	0.664	2.402	0.798, 6.881	0.121
Miscarriage	2.006	0.266, 1.238	0.157	3.757	0.128, 1.012	0.053
**Flexible protocol**	Coefficient	t	*P*	Coefficient	t	P
OPU number	0.000	-0.023	0.982	-0.034	-1.444	0.149
Number of usable embryos	0.031	1.576	0.115	0.041	1.826	0.068
Number of good quality embryos	0.023	0.979	0.328	0.044	1.538	0.124
	Wald value	95% CI	*P*	Wald value	95% CI	*P*
OHSS	0.076	0.516, 1.648	0.783	0.163	0.555, 2.444	0.686
Clinical pregnancy	0.521	0.679, 1.196	0.470	0.820	0.610, 1.199	0.365
Live birth	0.296	0.695, 1.228	0.587	0.247	0.420, 1.677	0.619
Multiple pregnancy	1.273	0.783, 2.485	0.259	0.027	0.457, 1.940	0.942
Miscarriage	0.260	0.656, 2.048	0.610	0.307	0.630, 2.286	0.580

PSM, propensity score matching; OPU, oocytes pick up; OHSS, ovarian hyper-stimulation syndrome; 95% CI, 95% confidential interval.

## 4 Discussion

ART services have shown a continuous growth trend in recent years. According to statistics released by the International Committee for Monitoring Assisted Reproductive Technologies (ICMART) in 2022, a total of 3.19 million ART cycles were reported globally, with 1.07 million occurring in China (13). Considering the increasing demand for ART services, effective, safe, and financially viable treatment options are highly needed. Particularly, high treatment burden stands as a critical factor leading to ART treatment discontinuation and poor treatment experiences (14).

The GnRH antagonist protocol stands as the predominant protocol for Controlled Ovarian Stimulation (COS) worldwide. According to the Deutsches IVF Register Annual Report, more than 77.5% of patients underwent COS using the GnRH antagonist protocol, in contrast to only 14.5% who received the GnRH agonist protocol (15). In China, the use of GnRH antagonist regimens for COS has increased substantially, rising from 6% in 2014 to 37% in 2021 (16). The GnRH antagonist is a crucial component of this protocol. Preclinical studies of GnRH antagonists have shown no detrimental effects on the fetus, no mutagenic or teratogenic impacts on the human body. GnRH antagonists exhibit comparable implantation rates, clinical pregnancy rates, and live birth rates, with a lower risk of OHSS compared to GnRH agonists (17).

Cetrotide^®^ stands as one of the initial GnRH antagonist preparations approved by the European Medicines Agency (EMA) in Europe. It is used to prevent premature ovulation as part of COS treatment by inhibiting LH secretion (18). The generic product is nearly identical to an existing EMA-approved reference product, showing no meaningful differences in terms of clinical efficacy, side effects, and immunogenicity (19). Fewer clinical trials are required compared to the reference biologics, significantly reducing the cost of generics (19, 20). Despite of the near interchangeability of generics and reference biologic products, the benefit-risk profiles of generics remain unclear due to limited pre-marketing trials on efficacy and safety information (20). Ferpront^®^ is the first generic of Cetrotide^®^ in China and has been utilized in numerous major reproductive centers across the country since its market introduction. However, there is a lack of clinical data regarding the efficacy and safety of Ferpront^®^. To address clinicians’ needs for evidence-based information, we introduce one of the first piece of real-world evidence to compare the clinical efficacy and safety between generic Ferpront^®^ and its original product Cetrotide^®^.

This comparability study involving the two types of GnRH antagonist extends the understanding of the therapeutic efficacy and safety of GnRH antagonists. Here, we have demonstrated the therapeutic equivalence of Ferpront^®^ and Cetrotide^®^ in controlled ovarian stimulation and reproductive outcomes in infertile women undergoing IVF/ICSI using the GnRH antagonist protocol. Patients receiving these two types of GnRH antagonists showed comparable numbers of 2PN embryos, usable embryos, and good-quality embryos on day 3, as well as similar incidences of moderate/severe OHSS, clinical pregnancy rates, miscarriage rates, and live birth rates.

The present study is powered by the primary outcome, live birth rate, one of the most critical objectives of ART therapy. Other reproductive outcomes, including clinical pregnancy rate and miscarriage rate, were also similar between the two types of GnRH antagonists. The study findings revealed that women in group B yielded similar reproductive outcomes compared to those in group A concerning embryo implantation, clinical pregnancy, miscarriage, and live birth rates. The duration of GnRH antagonist in group B is a little longer than that of group A (5.72 ± 1.47 vs. 6.01 ± 1.42 days before matching, and 5.71 ± 1.48 vs. 6.02 ± 1.41 days after matching), but the differences showed minimal clinical values. However, we observed approximately 0.8 fewer oocytes retrieved in cycles with group B than in group A (14.17 ± 7.30 vs. 14.96 ± 7.75 oocytes). Notably, women in group A showed higher levels of serum E2 on trigger day than in group B, which could probably lead to more yield of oocytes (21). Although the precise reasons remain unknown, this phenomenon likely holds little clinical significance, given that the fertilization and cleavage rates, as well as the amounts of usable embryos and good-quality embryos on day 3, remained equivalent between the two groups. Multivariate linear regression analysis showed no obvious impact of the type of GnRH antagonist on the number of oocytes retrieved. The duration of GnRH antagonist usage in group B was 0.3 days longer than in group A. Although statistically significant, this difference revealed minimal clinically substantive value, especially when considering the nearly equivalent duration and dose of Gn and the dose of GnRH antagonist. These results were further supported by data from propensity score matching, demonstrating similar effectiveness and safety of the two types of GnRH antagonist.

Likewise, other studies have also demonstrated that most generics/biosimilars do not significantly differ from their originators. For instance, follitropin alfa original (Gonal-f^®^) and generic (Ovaleap^®^) showed similar safety and efficacy in infertile ovulatory women undergoing ART (22). Hu et al (23) reported an equivalent effect of Gonal-f^®^ and its generic QL1012. Although there are few reports of GnRH antagonist generics available at present, we believe that there is no significant difference between the generics and the original product of GnRH antagonist.

This study represents one of the initial comparative examinations between Ferpront^®^ and Cetrotide^®^. It encompassed a substantial number of participants across a wide spectrum of infertile couples. The primary focus on live birth rate as the key endpoint aligns with one of the pivotal goals in assisted reproduction. The inclusion of a relatively large sample from one of the most voluminous reproductive centers, characterized by a standardized treatment regimen, bolsters the study’s credibility. Moreover, to mitigate potential selection biases and confounding factors, PSM and multivariate regression analyses were conducted to assess the independent impact of the GnRH antagonist type, further enhancing the solidity of the results. A limitation lies in the retrospective design, and several parameters including the number of mature oocytes retrieved was not analyzed, and possible selection bias cannot be avoided completely. However, despite of this aspect, this real-world study provides valuable insights into the effectiveness and safety of these treatments in routine ART practice.

## 5 Conclusion

This study supports the conclusion that there are no clinically significant differences between Ferpront^®^ and Cetrotide^®^ concerning clinical efficacy and safety when used in GnRH antagonist protocols for COS. The study’s results indicate the therapeutical equivalence and safety alignment of Ferpront^®^ and Cetrotide^®^.

## Data availability statement

The raw data underlying this article will be shared on reasonable request to the corresponding author.

## Ethics statement

The studies involving humans were approved by the local hospital ethics committee (Approval number is 2023-121). The studies were conducted in accordance with the local legislation and institutional requirements. The participants provided their written informed consent to participate in this study.

## Author contributions

MC: Data curation, Formal analysis, Funding acquisition, Writing – original draft, Writing – review & editing. YH: Formal analysis, Writing – review & editing. JX: Formal analysis, Writing – review & editing. SL: Data curation, Funding acquisition, Writing – review & editing. YL: Data curation, Writing – review & editing. JL: Data curation, Funding acquisition, Investigation, Writing – review & editing. HL: Conceptualization, Project administration, Supervision, Writing – review & editing.

## Supplementary material

Supplementary Table 1 SMD of ovarian reserve markers before and after PSM. Denotes; PSM, propensity score matching; SMD, standardized mean difference; n, number; AMH, anti-Mullerian hormone; FSH, follicular stimulating hormone; AFC, antral follicle counting.
