# Supplementary material for: Effectiveness and safety of GnRH antagonist originator and generic in real-world clinical practice: a retrospective cohort study
Source: Front Endocrinol (Lausanne). 2024 Jun 14;15:1358278. doi: 10.3389/fendo.2024.1358278 (PMC11211621; doi:10.3389/fendo.2024.1358278)
Supplement: Supplementary Table 1 — SMD of ovarian reserve markers before and after PSM. Denotes; PSM, propensity score matching; SMD, standardized mean difference; n, number; AMH, anti-Mullerian hormone; FSH, follicular stimulating hormone; AFC, antral follicle counting. [file Table_1.docx]

**Supplemental Table 1. SMD of ovarian reserve markers before and after PSM**

|  | Before PSM | | | |  | After PSM | | | | |
| --- | --- | --- | --- | --- | --- | --- | --- | --- | --- | --- |
|  | Group A | Group B | *P* | SMD |  | Group A | Group B | *P* | | SMD |
| n | 920 | 1977 |  |  |  | 915 | 915 |  | |  |
| AMH | 5.59 ± 4.13 | 5.22 ± 3.72 | 0.014 | 0.096 |  | 5.59 ± 4.13 | 5.48 ± 3.89 | 0.576 | 0.026 | |
| Basal FSH | 6.11 ± 2.39 | 5.91 ± 2.11 | 0.025 | 0.088 |  | 6.07 ± 2.29 | 6.03 ± 2.28 | 0.672 | 0.020 | |
| AFC | 22.97 ± 10.17 | 22.20 ±9.48 | 0.047 | 0.078 |  | 22.97 ± 10.17 | 22.99 ± 10.28 | 0.958 | 0.002 | |

Denotes; PSM, propensity score matching; SMD, standardized mean difference; n, number; AMH, anti-Mullerian hormone; FSH, follicular stimulating hormone; AFC, antral follicle counting.
